# Recombinant human soluble thrombomodulin for acute exacerbation of idiopathic pulmonary fibrosis: a nationwide observational study

**DOI:** 10.1186/s40560-022-00608-5

**Published:** 2022-03-09

**Authors:** Nobuyasu Awano, Taisuke Jo, Takehiro Izumo, Minoru Inomata, Kojiro Morita, Hiroki Matsui, Kiyohide Fushimi, Hirokazu Urushiyama, Takahide Nagase, Hideo Yasunaga

**Affiliations:** 1 Department of Respiratory Medicine, Japanese Red Cross Medical Center, 4-1-22 Hiroo, Shibuya-ku, Tokyo, 150-8935 Japan; 2 Department of Health Services Research, Graduate School of Medicine, The University of Tokyo, Tokyo, Japan; 3 Department of Respiratory Medicine, Graduate School of Medicine, The University of Tokyo, Tokyo, Japan; 4 Department of Clinical Epidemiology and Health Economics, School of Public Health, The University of Tokyo, Tokyo, Japan; 5 Department of Health Services Research, Faculty of Medicine, University of Tsukuba, Ibaraki, Japan; 6 Department of Health Policy and Informatics, Tokyo Medical and Dental University Graduate School of Medicine, Tokyo, Japan

**Keywords:** Idiopathic pulmonary fibrosis, Steroids, Recombinant human soluble thrombomodulin, Propensity score, Mortality

## Abstract

**Background:**

Acute exacerbation of idiopathic pulmonary fibrosis (AE-IPF) is the leading cause of death among patients with IPF. However, there is no established treatment for this condition. Hence, we aimed to investigate the effectiveness and safety of recombinant human soluble thrombomodulin (rTM) for the treatment of AE-IPF.

**Methods:**

Data were retrospectively collected from the Japanese Diagnosis Procedure Combination database from 1 January 2014 to 31 March 2018. We identified adult patients with IPF who received high-dose methylprednisolone (mPSL) therapy and mechanical ventilation upon admission. Eligible patients (*n* = 2814) were divided into those receiving high-dose mPSL alone (mPSL alone group, *n* = 2602) and rTM combined with high-dose mPSL (rTM group, *n* = 212). A stabilised inverse probability of treatment weighting (IPTW) using propensity scores was performed to compare outcomes between the two groups. The primary outcome was in-hospital mortality, and the secondary outcomes were 14- and 28-day mortality, bleeding events and length of hospital stay.

**Results:**

The in-hospital mortality rates of the mPSL alone and rTM groups were 75.9% and 76.9%, respectively. The results did not significantly differ between the two groups after performing a stabilised IPTW. The odds ratio of the rTM group compared to the mPSL alone group was 1.15 (95% confidence interval: 0.71–1.84; *p* = 0.57). Moreover, the secondary outcomes did not differ significantly between the two groups.

**Conclusions:**

In patients with AE-IPF who developed severe respiratory failure, rTM in addition to high-dose mPSL was not associated with a better outcome.

**Supplementary Information:**

The online version contains supplementary material available at 10.1186/s40560-022-00608-5.

## Background

Idiopathic pulmonary fibrosis (IPF) is an interstitial lung disease characterised by chronic fibrosis. In addition, it has a poor prognosis, with an average survival time of 3–4 years [1]. A previous study showed that acute exacerbation of IPF (AE-IPF) was the leading cause of death among patients with IPF. Moreover, it was correlated with a high mortality, with a mean survival time of < 1 year and a 90-day mortality rate of approximately 50% [2].

There is no established treatment for AE-IPF. Based on the Japanese and international guidelines, the therapeutic options include immunosuppressive agents and corticosteroids, such as high-dose methylprednisolone (mPSL) [3, 4]. Although the etiology and pathobiology of AE-IPF remain unclear, it is speculated that AE-IPF is associated with pulmonary vascular endothelial cell injury caused by inflammation and impaired pulmonary microcirculation attributed to microthrombi induced by coagulopathy [5–8]. Thrombomodulin is a transmembrane glycoprotein present on the endothelial cell surface of the body, and it plays an important role in regulating coagulation cascade [9]. Recombinant human soluble thrombomodulin (rTM) (Recomodulin, Asahi Kasei Pharma Co., Tokyo, Japan) exhibits a range of physiologically important anticoagulant, antifibrinolytic and anti-inflammatory properties [10]. Thus, it may be effective for the treatment of AE-IPF. In fact, several studies revealed that rTM improved the short-term prognosis of AE-IPF [11–14]. However, all these studies were performed at single institutions and had small numbers of patients. Furthermore, a recent double-blind randomised controlled trial did not show the efficacy of rTM in patients with mild AE-IPF who did not require mechanical ventilation [15]. Taken together, the effectiveness of rTM in patients with AE-IPF who develop severe respiratory failure requiring mechanical ventilation remains unclear.

The current study aimed to evaluate the effectiveness and safety of rTM in patients with AE-IPF who developed severe respiratory failure using data collected from a Japanese nationwide inpatient database. In addition, the incidence of bleeding events and length of hospital stay were investigated.

## Methods

### Data source

Inpatient data were extracted from the Japanese Diagnosis Procedure Combination database, the details of which have been reported elsewhere [16]. More than 1000 hospitals voluntarily contributed to the database, representing approximately 50% of all discharges from acute care hospitals in Japan. We collected data including those of sex and age; hospitalisation and discharge dates; weight and height; severity of dyspnoea based on the Hugh–Jones dyspnoea scale [17]; level of consciousness upon admission; smoking index; activities of daily living; frequency of hospitalisation; intensive care unit (ICU) admission during hospitalisation; main diagnoses, pre-existing comorbidities upon admission and complications after admission as recoded by the attending physicians based on the International Classification of Diseases, 10th revision (ICD-10) codes accompanied by text in Japanese; procedures and their dates; dates and doses of drugs administered during hospitalisation; and discharge status.

This study was approved by the institutional review board of The University of Tokyo. The need for informed consent was waived, because anonymised data were used.

### Patient selection

This study used data collected from 1 January 2014 to 31 March 2018. The inclusion criteria were patients aged ≥ 15 years, those diagnosed with interstitial pneumonia (ICD-10 codes J84.1, J84.8 and J84.9), those who underwent computed tomography scan within 1 day after admission and those who received treatment with intravenous mPSL at a dose of 500–1000 mg/day for 3 days, which was started within 4 days after admission [18, 19]. Patients with IPF were selected as follows. Initially, patients with idiopathic interstitial pneumonias other than IPF, such as idiopathic nonspecific interstitial pneumonia, respiratory bronchiolitis-associated interstitial lung disease, cryptogenic organizing pneumonia, acute interstitial pneumonia, desquamative interstitial pneumonia, lymphoid interstitial pneumonia, idiopathic pleuroparenchymal fibroelastosis and unclassifiable idiopathic interstitial pneumonia, were excluded using the diagnoses in Japanese. Moreover, we did not include patients with secondary interstitial lung diseases identified using ICD-10 codes (hypersensitivity pneumonitis [J67], connective tissue disease associated with interstitial lung disease [M05, M06 and M30–35], sarcoidosis [D86], amyloidosis [E85], drug-induced lung disease [J70], radiation pneumonitis [J70], *Pneumocystis jirovecii* pneumonia [B59], pneumoconiosis [J60–65], pulmonary alveolar proteinosis [J84.0] eosinophilic pneumonia [J82], Langerhans cell histiocytosis [C96] and lymphangioleiomyomatosis [D21.9]; those receiving medications including furosemide, azosemide, carperitide, landiolol hydrochloride, digoxin, deslanoside and tolvaptan for acute heart failure within 1 day after admission; and those who received intra-aortic balloon pump therapy during hospitalisation [18, 19]. The remaining patients were assumed to have AE-IPF. Next, we excluded patients with missing data about treatment year, those without mechanical ventilation and those who died within 6 days after admission to prevent immortal time bias. Patients were divided into two groups: those who received high-dose mPSL alone (mPSL alone group) and those who received rTM for at least 3 days, which was started within 4 days after admission, combined with high-dose mPSL (rTM group).

### Characteristics of patients

The characteristics of patients evaluated in this study were sex, age, treatment year, body mass index, Hugh–Jones dyspnoea scale scores upon admission, level of consciousness upon admission, comorbidities, Charlson Comorbidity Index, smoking index, activities of daily living scale (Barthel Index) upon admission, history of previous hospitalisation (0, 1–2, or ≥ 3), type of hospital (academic or non-academic hospital) and ICU admission. Furthermore, we examined data about procedures and treatments, including mechanical ventilation, continuous renal replacement therapy, high-flow nasal cannula oxygen therapy, transfusion and use of medications for IPF within 3 days after admission. Level of consciousness upon admission was evaluated using the Japan Coma Scale, [20, 21] which is widely used in Japan and is well correlated with the Glasgow Coma Scale score [22]. The following comorbidities were identified using ICD-10 codes (Additional file 1: Table S1): bronchial asthma, pulmonary emphysema, pneumonia, mycotic infection, pulmonary embolism, bronchiectasis, pneumothorax, cor pulmonale, lung and other types of cancer, sepsis, chronic heart failure, tachycardia, acute coronary syndrome, diabetes mellitus, stroke, dementia, renal failure, liver dysfunction and gastroesophageal reflux disease. The Charlson Comorbidity Index scores were classified into four categories (0, 1, 2 and ≥ 3).

### Outcome

The primary outcome was all-cause in-hospital mortality. The secondary outcomes were 14- and 28-day mortality, post-hospitalisation bleeding events and length of hospital stay. The following bleeding events were assessed using ICD-10 codes: epistaxis (R040), hemoptysis (R042), pulmonary hemorrhage (R048), subcutaneous hemorrhage (R233), purpura (D692), muscle hemorrhage (T146), hematuria (R31), gastrointestinal bleeding (K228, K922), bloody stool (K921) and intracranial hemorrhage (I61, I629) [15].

### Statistical analysis

Dichotomous and categorical variables were presented as numbers with percentages and continuous variables as median and interquartile range (IQR).

To account for differences in baseline characteristics, including comorbidities and treatments, stabilised inverse probability of treatment weighting (IPTW) analyses using propensity scores were performed to compare outcomes between the two groups. Stabilised IPTW uses propensity scores and adjusts for measured potential confounders while preserving sample size [23]. To control covariate imbalance, the specific stabilised weights were generated using propensity scores, which can predict the probability of receiving rTM combined with high-dose mPSL therapy. To estimate the propensity score, a logistic regression model for receiving high-dose mPSL alone therapy was used with the following independent variables: sex, age, treatment year, body mass index, Hugh–Jones dyspnoea scale score, level of consciousness upon admission, Charlson Comorbidity Index, smoking index, Barthel Index upon admission, frequency of hospitalisation, ICU hospitalisation within 3 days after admission, comorbidities and procedures (hemodialysis, high-flow nasal cannula oxygen therapy, fresh frozen plasma transfusion and concentrated platelet transfusion) and drugs for AE-IPF and disseminated intravascular coagulation (DIC) (noradrenaline, azithromycin, cyclophosphamide, cyclosporin, tacrolimus, azathioprine, pirfenidone, nintedanib, sivelestat sodium hydrate, heparin calcium, dalteparin and tranexamic acid). Variables included in the logistic regression model were those that were considered as potential confounders with reference to previous studies [18, 19]. Covariate balance was assessed using a standardised mean difference. A value of < 0.20 indicated an acceptable balancing of covariates between the two groups. Stabilised IPTW analyses can preserve sample size and appropriately estimate average treatment effects over the marginal distribution of measured covariates in a study cohort.

We used generalised linear models with cluster-robust standard errors treating each hospital as a cluster to compare the primary and secondary outcomes. Logistic regression analyses of in-hospital mortality, 14- and 28-day mortality and post-hospitalisation bleeding events were conducted. Then, odds ratios and their 95% confidence intervals (CIs) were calculated. The lengths of hospital stay between the two groups were compared via Poisson regression analysis, and the incidence rate ratios and their 95% CIs were calculated. To address competing outcomes, secondary outcomes were evaluated among the survivors alone and all patients.

A two-tailed significance level of 0.05 was used in all statistical analyses. All tests were performed using STATA/MP version 16 software (STATA Corp., College Station, TX, USA).

## Results

**Fig. 1:**
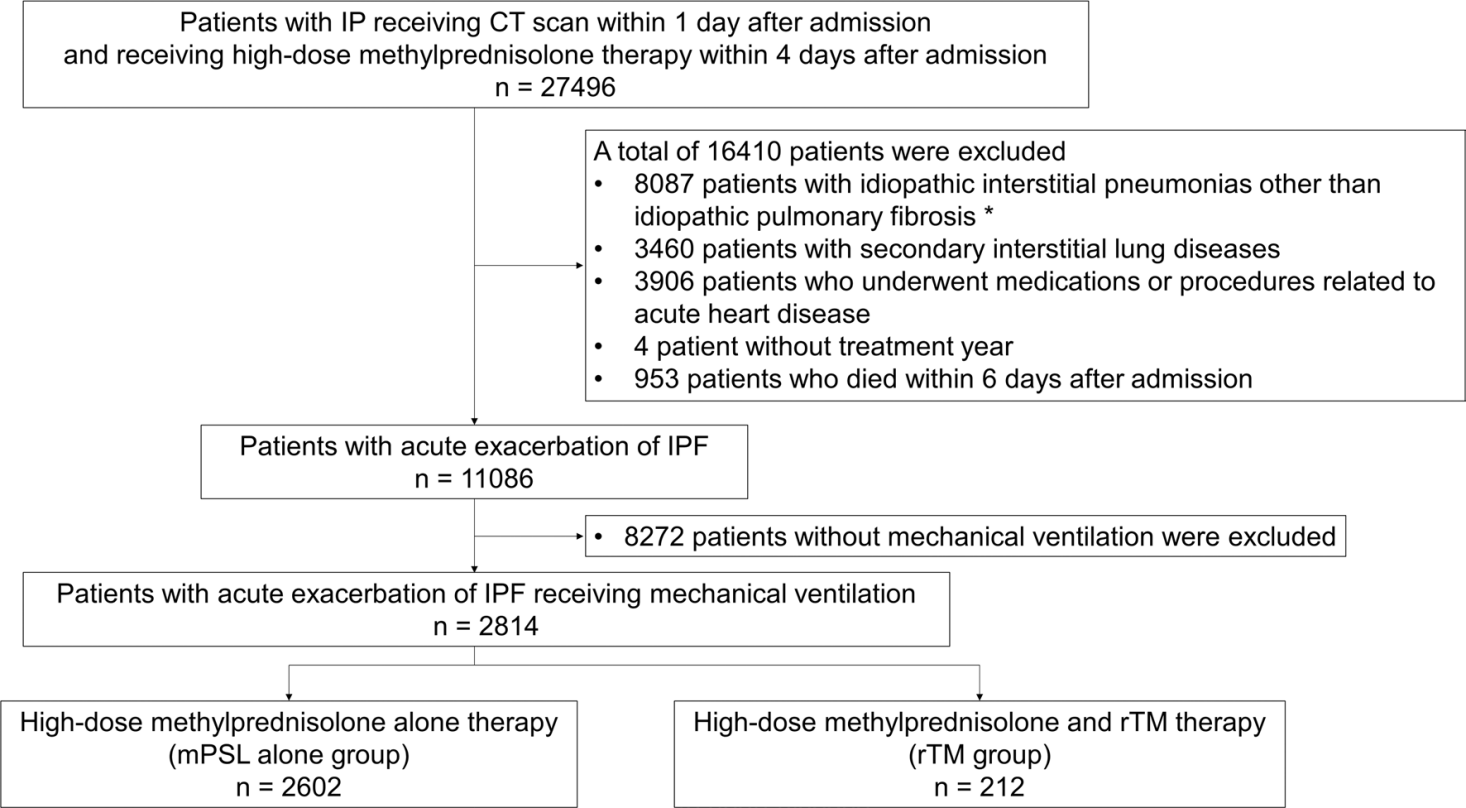
Flow chart of patient selection. *Idiopathic nonspecific interstitial pneumonia, respiratory bronchiolitis-associated interstitial lung disease, cryptogenic organizing pneumonia, acute interstitial pneumonia, desquamative interstitial pneumonia, lymphoid interstitial pneumonia, idiopathic pleuroparenchymal fibroelastosis and unclassifiable idiopathic interstitial pneumonia. *IP* interstitial pneumonia, *CT* computed tomography, *IPF* idiopathic pulmonary fibrosis, *mPSL* methylprednisolone, *rTM* recombinant human soluble thrombomodulin

Figure 1 depicts the process of patient selection. During the study period, 27,496 patients underwent computed tomography scan within 1 day and received high-dose mPSL corticosteroid therapy within 4 days after admission. Among them, 2814 were eligible for this study. The patients were divided into the mPSL alone group (*n* = 2602) and the rTM group (*n* = 212).

Table 1 shows the baseline characteristics of patients, and Table 2 presents comorbidities and treatments before and after stabilised IPTW. The mPSL alone group had a higher proportion of patients aged older than 80 years than the rTM group. Moreover, the Hugh–Jones dyspnoea scale scores were not balanced between the two groups. The rTM group had a higher proportion of patients who received azithromycin, cyclophosphamide, tacrolimus, pirfenidone, sivelestat sodium hydrate and concentrated platelet transfusion than the mPSL alone group. Meanwhile, the mPSL alone group had a higher percentage of patients who received heparin calcium than the rTM group. Less than 5% of patients in both two groups received heparin calcium, dalteparin, tranexamic acid and blood transfusions. After the stabilised IPTW using propensity scores, the baseline characteristics of the patients were well balanced between the two groups.

**Table 1:** Baseline characteristics of patients before and after the stabilised IPTW using propensity scores

Characteristics	All patients					Patients after IPTW estimation				
	mPSL alone group (*n* = 2602)	%	rTM group (*n* = 212)	%	SMD	mPSL alone group (*n* = 2611)	%	rTM group (*n* = 190)	%	SMD
Male sex	1962	75.4	159	75	− 0.9	1971	75.5	137	72.1	− 7.6
Age, years										
15–70	640	24.6	64	30.2	12.6	655	25.1	54	28.4	8.2
71–80	1147	44.1	104	49.1	10	1159	44.4	91	47.9	6.7
≥ 80	805	30.9	43	20.3	− 24.6	787	30.1	43	22.6	− 17.2
Missing data	10	0.4	1	0.5	1.3	10	0.4	2	1.1	7.3
Treatment year										
2014	546	21	38	17.9	− 7.7	541	20.7	36	18.9	− 4.6
2015	566	21.8	60	28.3	15.2	583	22.3	41	21.6	− 1.7
2016	531	20.4	43	20.3	− 0.3	532	20.4	41	21.6	3.7
2017	472	18.1	42	19.8	4.3	475	18.2	29	15.3	− 7.6
2018	487	18.7	29	13.7	− 13.7	482	18.5	42	22.1	9.7
BMI (kg/m^2^)										
< 23	1282	49.3	105	49.5	0.5	1288	49.3	102	53.7	9.1
23–25	507	19.5	35	16.5	− 7.8	500	19.1	29	15.3	− 10.2
≥ 25	555	21.3	52	24.5	7.6	567	21.7	43	22.6	1.9
Missing data	258	9.9	20	9.4	− 1.6	257	9.8	16	8.4	− 5.2
Hugh–Jones dyspnoea score upon admission										
1–2	205	7.9	15	7.1	− 3.1	202	7.7	13	6.8	− 3.2
3	168	6.5	8	3.8	− 12.2	163	6.2	11	5.8	− 2.7
4	329	12.6	23	10.8	− 5.6	324	12.4	18	9.5	− 9.7
5	1177	45.2	69	32.5	− 26.2	1154	44.2	85	44.7	0.7
Missing data	723	27.8	97	45.8	37.9	767	29.4	64	33.7	9
Japan Coma Scale score upon admission										
0- or 1-digit (alert or dull)	2460	94.5	195	92	− 10.2	2463	94.3	180	94.7	2.7
2-digit (somnolence)	73	2.8	9	4.2	7.8	77	2.9	4	2.1	− 3.8
3-digit (coma)	69	2.7	8	3.8	6.4	72	2.8	5	2.6	0
Charlson Comorbidity Index										
0	545	20.9	45	21.2	0.7	544	20.8	39	20.5	− 0.2
1	357	13.7	27	12.7	− 2.9	358	13.7	28	14.7	2.7
2	510	19.6	34	16	− 9.3	504	19.3	32	16.8	− 5.7
≥ 3	303	11.6	21	9.9	− 5.6	302	11.6	20	10.5	− 3.0
Missing data	887	34.1	85	40.1	12.5	904	34.6	70	36.8	4.7
Smoking index, pack-years										
0	1113	42.8	79	37.3	− 11.3	1104	42.3	81	42.6	0.5
1–39	546	21	50	23.6	6.3	553	21.2	37	19.5	− 4.8
≥ 40	618	23.8	51	24.1	0.7	618	23.7	37	19.5	− 10.8
Missing data	325	12.5	32	15.1	7.6	337	12.9	36	18.9	16.7
ADL upon admission (Barthel Index)										
100	519	19.9	53	25	12.1	526	20.1	33	17.4	− 6.6
≤ 95	1612	62	118	55.7	− 12.8	1607	61.5	120	63.2	3.7
Missing data	471	18.1	41	19.3	3.2	479	18.3	36	18.9	2.0
History of previous hospitalisation										
0	1457	56	110	51.9	− 8.2	1455	55.7	108	56.8	2.4
1–2	792	30.4	78	36.8	13.5	807	30.9	57	30.0	− 1.9
≥ 3	353	13.6	24	11.3	− 6.8	350	13.4	25	13.2	− 0.9
Academic hospital	2169	83.4	179	84.4	2.9	2179	83.5	164	86.3	7.6
ICU admission	665	25.6	68	32.1	14.4	683	26.2	55	28.9	6.3

Data were presented as *n* (%)

*BMI* body mass index, *ADL* activities of daily living, *ICU* intensive care unit, *IPTW* inverse probability of treatment weighting, *mPSL* methylprednisolone, *rTM* recombinant human soluble thrombomodulin, *SMD* standardised mean difference

**Table 2:** Comorbidities and treatments before and after the stabilised IPTW using propensity scores

Variables	All patients					Patients after IPTW estimation				
	mPSL alone group (*n* = 2602)	%	rTM group (*n* = 212)	%	SMD	mPSL alone group (*n* = 2611)	%	rTM group (*n* = 190)	%	SMD
Comorbidity										
Bronchial asthma	141	5.4	13	6.1	3.1	141	5.4	7	3.7	− 8.2
Pulmonary emphysema	101	3.9	7	3.3	− 3.1	99	3.8	5	2.6	− 7.1
Pneumonia	488	18.8	48	22.6	9.6	500	19.1	36	18.9	− 0.4
Mycotic infection	16	0.6	6	2.8	17.1	22	0.8	2	1.1	0.2
Pulmonary embolism	14	0.5	0	0	− 10.4	15	0.6	0	0.0	− 10.8
Bronchiectasis	79	3.0	6	2.8	− 1.2	78	3.0	4	2.1	− 3.8
Pneumothorax	23	0.9	2	0.9	0.6	23	0.9	1	0.5	− 2.0
Cor pulmonale	27	1.0	5	2.4	10.2	29	1.1	2	1.1	0.9
Lung cancer	214	8.2	20	9.4	4.3	218	8.3	13	6.8	− 6.2
Other types of cancer^a^	187	7.2	12	5.7	− 6.2	184	7.0	10	5.3	− 6.7
Sepsis	59	2.3	10	4.7	13.4	67	2.6	12	6.3	19.3
Chronic heart failure	362	13.9	23	10.8	− 9.3	358	13.7	22	11.6	− 6.4
Tachycardia	186	7.1	12	5.7	− 6.1	183	7.0	11	5.8	− 5.9
Acute coronary syndrome	171	6.6	11	5.2	− 5.9	167	6.4	4	2.1	− 17.6
Diabetes mellitus	681	26.2	51	24.1	− 4.9	676	25.9	43	22.6	− 6.9
Stroke	146	5.6	9	4.2	− 6.3	143	5.5	9	4.7	− 2.7
Dementia	66	2.5	2	0.9	− 12.2	63	2.4	2	1.1	− 12.0
Renal failure	272	10.5	27	12.7	7.1	281	10.8	18	9.5	− 4.7
Liver dysfunction	123	4.7	10	4.7	0	123	4.7	15	7.9	15.9
Gastroesophageal reflux disease	339	13.0	23	10.8	− 6.7	335	12.8	24	12.6	− 0.8
Treatment within 3 days after hospitalisation										
Noradrenaline	21	0.8	4	1.9	9.4	24	0.9	2	1.1	3.1
Azithromycin	520	20.0	71	33.5	30.8	547	20.9	34	17.9	− 6.9
Cyclophosphamide (intravenous)	61	2.3	14	6.6	20.7	72	2.8	7	3.7	4.5
Cyclosporin	88	3.4	15	7.1	16.6	98	3.8	11	5.8	9.2
Tacrolimus	27	1.0	18	8.5	35.5	42	1.6	3	1.6	0.2
Azathioprine	9	0.3	0	0	− 8.3	9	0.3	0	0.0	− 8.2
Pirfenidone	49	1.9	18	8.5	30.1	62	2.4	4	2.1	− 0.4
Nintedanib	32	1.2	7	3.3	13.9	35	1.3	4	2.1	4.4
Sivelestat sodium hydrate	341	13.1	72	34.0	50.6	385	14.7	32	16.8	5.7
Heparin calcium (intravenous)	52	2.0	0	0	− 20.2	51	2.0	0	0.0	− 19.9
Dalteparin	20	0.8	1	0.5	− 3.8	19	0.7	0	0.0	− 7.7
Tranexamic acid	95	3.7	2	0.9	− 18.1	90	3.4	4	2.1	− 8.3
Haemodialysis	96	3.7	14	6.6	13.2	104	4.0	11	5.8	7.6
High-flow nasal cannula oxygen therapy	164	6.3	22	10.4	14.8	172	6.6	13	6.8	0.7
Fresh frozen plasma transfusion	25	1.0	8	3.8	18.5	32	1.2	7	3.7	16.4
Concentrated platelet transfusion	21	0.8	9	4.2	22.0	28	1.1	3	1.6	3.2

Data were presented as *n* (%)

*IPTW* inverse probability of treatment weighting, *mPSL* methylprednisolone, *rTM* recombinant human soluble thrombomodulin, *SMD* standardised mean difference

^a^Detailed information in Additional file 1: Table S1

The in-hospital mortality rates before the stabilised IPTW in the mPSL alone and rTM groups were 75.9% (1976/2602) and 76.9% (163/212), respectively. Table 3 presents the outcomes after the stabilised IPTW. The in-hospital mortality rates of the mPSL alone and rTM groups were 76.2% (1990/2611) and 78.4% (149/190), respectively. Table 4 shows the comparison of the outcomes of the mPSL alone and rTM groups after the stabilised IPTW. The results did not significantly differ between the two groups, and the odds ratio of the rTM group was 1.15 (95% CI 0.71–1.84; *p* = 0.57). Similarly, the odds ratios of 14- and 28-day mortality in the rTM group were 0.80 (95% CI 0.48–1.32; *p* = 0.38) and 0.79 (95% CI 0.53–1.19; *p* = 0.26), respectively. The proportions of patients with post-hospitalisation bleeding events were similar between the two groups, and the odds ratio of the rTM group was 1.60 (95% CI 0.70–3.64; *p* = 0.27). In the rTM group, the incidence rate ratio of length of hospital stay was 1.18 (95% CI 0.93–1.50; *p* = 0.18) compared with the mPSL alone group. In addition, there were no significant differences in terms of other secondary outcomes between the survivors of the two groups.

**Table 3:** Outcomes between the mPSL alone and rTM groups after the stabilised IPTW

	mPSL alone group	rTM group
All patients, (*n*)	2611	190
In-hospital mortality, *n* (%)	1990 (76.2)	149 (78.4)
14-day mortality, *n* (%)	746 (28.6)	46 (24.2)
28-day mortality, *n* (%)	1382 (52.9)	90 (47.4)
Bleeding events^a^	73 (2.8)	8 (4.2)
Length of hospital stay (days), median (IQR)	22 (13–42)	27 (14–42)
Survivor, (*n*)	622	41
Bleeding events^a^	12 (1.9)	0 (0)
Length of hospital stay (days), median (IQR)	41 (24–64)	50 (32–78)

*IQR* interquartile range, *mPSL* methylprednisolone, *rTM* recombinant human soluble thrombomodulin

^a^Bleeding events included epistaxis, hemoptysis, pulmonary hemorrhage, subcutaneous hemorrhage, purpura, muscle hemorrhage, hematuria, gastrointestinal bleeding, bloody stool and intracranial hemorrhage

**Table 4:** Comparison of outcomes between the mPSL alone and rTM groups after the stabilised IPTW

	Odds ratio^b^	95% CI	*p* value
Logistic regression analyses of patients in the rTM and mPSL alone groups after the stabilised IPTW			
All patients			
In-hospital mortality	1.15	0.71–1.84	0.57
14-day mortality	0.80	0.48–1.32	0.38
28-day mortality	0.79	0.53–1.19	0.26
Bleeding events^a^	1.60	0.70–3.64	0.27
Survivors			
Bleeding events^a^	0.33	0.04–2.68	0.30

	Incidence rate ratio^c^	95% CI	*p* value
Incidence rate ratios of length of hospital stay in the rTM and mPSL alone groups after the stabilised IPTW			
All patients			
Length of hospital stay	1.18	0.93–1.50	0.18
Survivors			
Length of hospital stay	1.34	0.95–1.92	0.098

*rTM* recombinant human soluble thrombomodulin, *mPSL* methylprednisolone, *IPTW* inverse probability of treatment weighting, *CI* confidence interval

^a^Bleeding events included epistaxis, hemoptysis, pulmonary hemorrhage, subcutaneous hemorrhage, purpura, muscle hemorrhage, hematuria, gastrointestinal bleeding, bloody stool and intracranial hemorrhage

^b^The odds ratio of the rTM group compared to the mPSL alone group

^c^The incidence rate ratio of the rTM group compared to the mPSL alone group

## Discussion

We examined the effectiveness of rTM combined with high-dose mPSL therapy in patients with AE-IPF using data from a nationwide inpatient database in Japan. Results showed no significant difference in terms of in-hospital mortality rate between the mPSL alone and rTM groups. Similarly, the 14- and 28-day mortality, frequency of post-hospitalisation bleeding events and length of hospital stay did not remarkably differ between the two groups.

Because disordered coagulation is involved in the pathogenesis of AE-IPF, anticoagulants can be an effective treatment option. A previous study with a small sample size showed that low-molecular weight heparin can improve AE-IPF survival [24]. To date, whether rTM is effective for the treatment of AE-IPF remains controversial. Kataoka et al. compared the outcomes of 20 patients with rTM-treated AE-IPF and 20 historical patients with AE-IPF. Results showed that the 3-month mortality of the rTM group (30.0%) was significantly lower than that of the control group (65.0%) [11]. Similarly, other retrospective studies using rTM for AE-IPF revealed a better short-term prognosis [12–14]. Moreover, a systematic review of these studies showed the beneficial effects of rTM among patients with AE-IPF [25]. However, due to the research design and small sample sizes, these studies did not perform a proper adjustment for the background characteristics of participants. Meanwhile, a randomised controlled study that examined the efficacy of rTM for AE-IPF failed to show an improvement in 90-day mortality and did not recommend the use of rTM for the treatment of AE-IPF [15]. Surprisingly, in their study, the 90-day survival rates of the rTM and placebo groups were 72.5% and 89.2%, respectively, which were extremely good. This can be possibly attributed to the fact that they excluded patients with severe conditions who received mechanical ventilation via intratracheal intubation, those who required treatment for DIC and those who had a history of AE-IPF. The mortality rate was significantly higher in both groups in the current study than in previous ones [11–15]. This is likely caused by the fact that our study included only patients with AE-IPF who developed severe respiratory failure and received mechanical ventilation. The international guidelines for the diagnosis and management of IPF had a weak recommendation against the use of mechanical ventilation for patients with respiratory failure caused by IPF [4]. However, in real-world clinical practice, several patients with AE-IPF who experienced deteriorated respiratory condition require mechanical ventilation. The effectiveness of rTM was not promising even in patients with AE-IPF who developed severe respiratory failure.

Considering the mechanism of action of rTM, bleeding can be a potential risk. However, the post-marketing surveillance data of rTM therapy in patients with sepsis complicated by DIC indicated that the incidence of bleeding events was low at 6.8% [26]. The incidence of bleeding events was extremely low in previous studies using rTM for the treatment of AE-IPF [11–15]. Similarly, in the current study, the incidence of bleeding events was extremely low, and the results did not significantly differ between the mPSL alone and rTM groups.

In Japan, rTM was approved for the treatment of DIC in 2008 due to its anticoagulant and anti-inflammatory effects. Since the current research included patients with AE-IPF who developed severe respiratory failure, it is likely that we might have included patients with AE-IPF complicated by DIC. To account for patients with DIC, we compared outcomes by balancing the two groups based on the use of treatments for DIC (heparin calcium, dalteparin, tranexamic acid and blood transfusion).

The strength of the current study was the large number of patients with AE-IPF that it included, compared with previous studies, which allowed for the adjustment of numerous measured confounders and comparison of hard outcomes, such as in-hospital mortality between the two groups. However, the unmeasured potential confounders, such as baseline pulmonary function, may have biased our results. This limitation of our study is owing to its retrospective design using the Japanese Diagnosis Procedure Combination database, which does not contain detailed clinical information. Nevertheless, taking into account both the strength and limitations, it appears that our study provided results that complement the results from a previous randomised controlled study, which did not show the advantages of rTM in patients with AE-IPF [15].

The current study had several limitations. First, because the database did not include data about laboratory examinations, pulmonary function test results, performance status and radiological findings, the severity of IPF at the onset of AE could not be accurately evaluated. We only included patients with mechanical ventilation to equalize the severity of AE-IPF between the two groups. In addition, baseline characteristics and treatments were well balanced between the two groups according to the stabilised IPTW. Second, although the diagnosis of IPF was made by a physician, it was not confirmed via radiological and pathological examinations. To accurately classify IPF, all cases of idiopathic interstitial pneumonias, other than IPF and secondary interstitial pneumonia, were excluded using the diagnoses in Japanese or ICD-10 codes, because the specificity of diagnoses in the database is high in general [27].

## Conclusions

In conclusion, for the treatment of patients with AE-IPF who developed severe respiratory failure, rTM combined with high-dose mPSL was not associated with a better in-hospital mortality.

## Supplementary Information

**Additional file 1: Table S1.** List of ICD-10 codes used to identify comorbidities.

## Data Availability

The data sets used and/or analysed during the current study are available from the corresponding author on reasonable request.

## Abbreviations

AE: Acute exacerbation
CI: Confidence interval
DIC: Disseminated intravascular coagulation
ICD-10: International Classification of Diseases, 10th revision
ICU: Intensive care unit
IPF: Idiopathic pulmonary fibrosis
IPTW: Inverse probability of treatment weighting
IQR: Interquartile range
mPSL: Methylprednisolone
rTM: Recombinant human soluble thrombomodulin
