# Supplementary material for: Recombinant human soluble thrombomodulin for acute exacerbation of idiopathic pulmonary fibrosis: a nationwide observational study
Source: J Intensive Care. 2022 Mar 9;10:14. doi: 10.1186/s40560-022-00608-5 (PMC8908698; doi:10.1186/s40560-022-00608-5)
Supplement: Supplementary file 1 — Additional file 1: Table S1. List of ICD-10 codes used to identify comorbidities. [file 40560_2022_608_MOESM1_ESM.docx]

**Additional file 1**

**Table S1.** List of ICD-10 codes used to identify comorbidities

| Bronchial asthma: J45, J46 |
| --- |
| Pulmonary emphysema: J43 |
| Pneumonia: A481, J100, J110, J12, J13, J14, J15, J16, J170, J178, J18, J85, J86 |
| Mycotic infection: A420, A43, B37, B380, B381, B382, B390, B391, B392, B400, B401, B402, B410, B420, B440, B441, B449, B460, J172 |
| Pulmonary embolism: I26 |
| Bronchiectasis: J40, J41, J42, J47 |
| Pneumothorax: J93, J94 |
| Cor pulmonale: I27 |
| Lung cancer: C34 |
| Other types of cancer: C00, C01, C02, C03, C04, C05, C06, C07, C08, C09, C10, C11, C12, C13, C14, C15, C16, C17, C18, C19, C20, C21, C22, C23, C24 |
| Sepsis: A40, A41 |
| Chronic heart failure: I50 |
| Tachycardia: I47, I48, I49, R000, T818 |
| Acute coronary syndrome: I20, I21, I22, I23, I24, I25 |
| Diabetes mellitus: E11 |
| Stroke: I60, I61, I62, I63, I64, I65, I66, I67, I68, I69 |
| Dementia: G30, F01, F03, F107 |
| Renal failure: E102, E112, E142, I120, N17, N18, N19 |
| Liver dysfunction: B89, B181, B182, B659, B661, K70, K71, K72, K73, K74, K76 |
| Gastroesophageal reflux disease: K21 |

ICD-10, the International Classification of Diseases, 10th revision
